# Microsurgical Clip Suspension to Prevent Optic Neuropathy Following Ligation of Anterior Communicating Artery Aneurysm: A Technical Report and Surgical Video

**DOI:** 10.7759/cureus.6354

**Published:** 2019-12-11

**Authors:** Douglas J Chung, Alejandro Matus, Vitaly Siomin

**Affiliations:** 1 Neurological Surgery, Florida International University Herbert Wertheim College of Medicine, Miami, USA; 2 Neurological Surgery, Miami Cancer Institute, Baptist Health South Florida, Miami, USA

**Keywords:** aneurysm clip, surgical technique, cerebral aneurysm, anterior communicating artery, optic nerve compression

## Abstract

Anterior communicating artery (ACoA) aneurysms are among the most common intracerebral aneurysms. Complications of ACoA aneurysm include subarachnoid hemorrhage, which may occur spontaneously or as a result of trauma. While prognosis of microsurgical clip ligation is excellent, iatrogenic afferent pupillary defect secondary to mechanical compression of the optic nerve by the clips is a known complication. Our report presents a case of a 59-year-old female status post resection of a pituitary macroadenoma one year ago with a three- to four-week history of progressively worsening headache found to have a 6.5 x 5.4 mm wide neck and irregularly dysplastic aneurysmal dilation of the ACoA. During the operation, two of the longer clips appeared to be touching the optic nerve and we utilized a clip suspension technique to relieve compression. This gently elevated and suspended the two clips up to the dura, allowing for a 2 mm gap between the optic nerve and clips. This maneuver relieved mechanical compression against the optic nerve and potentially mitigated the need for surgical re-exploration in the future.

## Introduction

Anterior communicating artery (ACoA) aneurysms are among the most common intracerebral aneurysms, where ruptured and unruptured cases account for 25%-38% of total cerebral aneurysm cases [1,2]. A common complication of ACoA aneurysm is subarachnoid hemorrhage (SAH), which may occur spontaneously or as a result of trauma [2]. Ruptured aneurysms cause approximately 85% of spontaneous SAHs, affecting nearly 30,000 people in the USA every year [3]. 

Unruptured intracranial aneurysms can be found in 2%-5% of the general population, with an increased prevalence in women and those over the age of 30 years [4]. ACoA aneurysms are characteristically clinically silent prior to rupture, at which point they may present with sudden headache and altered mental status [1]. The ACoA aneurysm has a propensity to hemorrhage, even at or below size limits considered safe for conservative management, especially in younger patients [4]. Although coil embolization has gained popularity in recent years over clipping, the BRAT trial and CARAT studies have shown superior durability of clipping to coiling, as well as decreased rates of recurrence [5]. 

The ACoA complex is adjacent to the hypothalamus, optic apparatus, and cognitive/emotional centers in the basal frontal lobes, while arteries emanating from the ACoA complex affect the basal ganglia, internal capsule, and motor/sensory cortex [4]. ACoAs are one of the most commonly seen ruptured aneurysms and tend to have greater associations with cognitive and behavioral deficits due to injury to the frontal cortex or striatum [6]. Additionally, visual deterioration and visual field defect may be seen with ACoA aneurysms by various mechanisms such as hemorrhage, aneurysm compression on the optic pathway, and poor blood circulation secondary to abnormalities in the optic pathway vessels [1]. Optic apparatus injuries may also occur due to compression from aneurysm clips; therefore, care should be taken that both temporary and permanent clips are placed in a trajectory that does not compress the visual structures [2].

Linzey et al. reported three cases of microsurgical clip ligation of the ACoA leading to afferent pupillary defect secondary to mechanical compression of the optic nerve by the clips [7]. These defects and visual field cuts required surgical re-exploration for correction in all three cases with repositioning of the aneurysm clips [7]. While near-complete restoration of vision was reported, the suture sling technique described in our technical report offers an intraoperative solution to circumvent the risks associated with re-operating immediately after the initial surgery.

## Technical report

History

A 59-year-old female status post resection of a pituitary macroadenoma one year ago presents with three- to four-week history of progressively worsening headache. Conventional angiography done to work-up headache revealed an incidental finding of a 6.5 x 5.4 mm wide neck and irregularly dysplastic aneurysmal dilation of the ACoA (Video 1 at 0:13). Other risk factors include arterial hypertension. 

**Video 1:** Microsurgical Clip Suspension - ACoA Aneurysm 0:02 - Introduction 0:13 - Preoerative Imaging 0:32 - Clip Placement 2:23 - Surgical Technique: Suture Sling 3:28 - Intraoperative IC Green Angiography 3:36 - Postoperative Imaging ACoA: Anterior Communicating Artery IC: Indocyanine

Operation

The patient underwent right frontotemporal craniotomy for clipping of the unruptured ACoA aneurysm with utilization of microscopic magnification and microsurgical techniques, neuro monitoring, and placement of left frontal ventriculostomy catheter. The procedure was performed under general anesthesia with endotracheal tube. 

We found an anterior inferiorly projecting, obliquely oriented, irregularly shaped, thin-walled, and wide-necked ACoA aneurysm with two daughter sacs. Technically there was no ACoA, the patient had a very large dominant right A1 that gave rise to the ipsilateral and contralateral A2. Contralateral A1 was very hypoplastic and joined the contralateral A2 past the aneurysm neck. 

Aneurysm was occluded in a picket-fence fashion, simultaneously reconstructing the contralateral A2 and dominant ipsilateral A1. Once the final clip is applied to secure the small separate lobule projecting superiorly (Video 1 at 1:18), the ACoA complex appears to be well preserved with complete occlusion of the aneurysm and good preservation of the parent vessels. Temporary clip was removed from the right A1 with good restoration of flow (Video 1 at 1:44). 

At this point, removal of the retractor demonstrates persistent compression of the right optic nerve by the two longer clips (Video 1 at 2:01). Considering the possibility of further worsening of the right optic nerve compression, we decided to proceed with suspension of the longer clips away from the optic apparatus through utilization of a suture sling. 

An 8-0 prolene stitch was passed through the dura, which served as an anchor for the suture sling (Video 1 at 2:28). Then, we carefully passed the needle through the aneurysm clips. This part has to be done carefully with the goal of avoiding strong pulling, otherwise risking dislodging the clips or causing aneurysm rupture. The suture is gradually tightened to widen the gap and in the end with this maneuver we achieved approximately a 2 mm gap between the optic nerve and clips (Video 1 at 2:42). The knot is tightened and the clips are gently elevated from the optic nerve, leaving them suspended. Excess suture is cut, and this results in a stable suture sling up to the dura of the frontal region. The optic nerve appears to be well decompressed and we felt we could close. 

Intraoperative indocyanine green angiography demonstrates complete obliteration of the ACoA aneurysm with preservation of the ACoA complex. Postoperative conventional angiography was performed as well, which demonstrated that the fusiform dilation of the ACoA is no longer visible with reconstruction of the vessel bifurcation and potency of all branches of the ACA (Video 1 at 3:36).

## Discussion

ACoA aneurysms present a special challenge during microsurgical treatment due to the anatomical variation of the anterior cerebral arteries, recurrent artery of Huebner, and the ACoA itself [8]. Large or complex aneurysms require different solutions such as reconstructing the parent vessel with multiple clips in a tandem technique or applying a parallel clip along the long axis of the aneurysm. The anatomy of the ACoA region is variable, and the close anatomical relationship between the aneurysm structure and optic nerve/chiasm must be considered when determining which approach to use. Our patient's complex fusiform aneurysm posed the risk of compromise to the lumen of the parent vessel with the use of a parallel clip, and therefore we decided to utilize multiple clips placed in a tandem technique or in a "picket fence" fashion. This technique is effectively utilized in many cases of challenging fusiform aneurysms. We subsequently utilized the suture sling technique to avoid afferent pupillary defect following microsurgical clipping of the ACoA aneurysm, secondary to mechanical compression by the clips. Different techniques related to this complication include the use of a bridging clip sutured onto the dura with a Gelfoam spacer. 

Several studies have demonstrated the efficacy of microsurgical clip ligation in the treatment of cerebral aneurysms through long-term follow-up angiography, with a 0.14% recurrence rate of nearly 700 clipped aneurysms without residua [9]. Bohnstedt et al. investigated 319 cases of ACoA aneurysms treated with microsurgical clip ligation [8]. Of these, 60 represented unruptured aneurysms and 259 were ruptured prior to treatment [8]. In this study, 92% of the unruptured ACoA aneurysms ultimately had an acceptable outcome with no mortality within this group. For those with ruptured aneurysms, the percentage of patients with an acceptable outcome after suffering SAH from ACoA aneurysm who underwent microsurgical clip ligation was 67.5% at the last follow-up. Additionally, Bohnstedt et al. found that patients with A1 aneurysms were more likely to develop symptomatic vasospasm at 41.7% compared to the entire group at 18.5%. This study also found statistically significant associations between inferior projecting ACoA aneurysms and younger patient age, high risk of rupture, and were less likely to require intraoperative digital subtraction angiography [8]. 

Despite the low recurrence rates of clipped ACoA aneurysms and favorable outcomes in those with unruptured ACoA aneurysms, complications may still occur. Additionally, patients may develop iatrogenic optic neuropathy secondary to clip compression after microsurgical aneurysm clip ligation [7]. 

## Conclusions

To our knowledge, this is the first report of utilizing a clip suspension technique via suture sling to prevent optic neuropathy status post microsurgical clip ligation of an ACoA aneurysm. While prognosis of microsurgical clip ligation is excellent, iatrogenic afferent pupillary defect secondary to mechanical compression of the optic nerve by the clips is a known complication. This technical report and surgical video present our intraoperative solution when persistent aneurysm clip compression of the optic nerve was encountered. Our maneuver elevated the clips away from the optic apparatus, relieving mechanical compression, and potentially mitigating the need for surgical re-exploration in the future. 

## References

[1] Park JH, Park SK, Kim TH, Shin JJ, Shin HS, Hwang YS (2009). Anterior communicating artery aneurysm related to visual symptoms. J Korean Neurosurg Soc.

[2] Kato Y, Nouri M, Shu G (2018). Surgery of anterior communicating artery aneurysms. Neurovascular Surgery.

[3] Sweeney K, Silver N, Javadpour M (2019). Subarachnoid haemorrhage (spontaneous aneurysmal). BMJ Clin Evid.

[4] Quiñones-Hinojosa A (2012). Schmidek & Sweet Operative Neurosurgical Techniques: Indications, Methods, and Results. 6th ed.

[5] Kim P, Jang SJ (2018). Management of recurrent cerebral aneurysm after surgical clipping: clinical article. J Korean Neurosurg Soc.

[6] Molyneux AJ, Kerr RS, Yu LM (2005). International subarachnoid aneurysm trial (ISAT) of neurosurgical clipping versus endovascular coiling in 2143 patients with ruptured intracranial aneurysms: a randomised comparison of effects on survival, dependency, seizures, rebleeding, subgroups, and aneurysm occlusion. Lancet.

[7] Linzey JR, Chen KS, Savastano L, Thompson BG, Pandey AS (2018). Optic neuropathy after anterior communicating artery aneurysm clipping: 3 cases and techniques to address a correctable pitfall. J Neurosurg.

[8] Bohnstedt BN, Conger AR, Edwards J (2019). Anterior communicating artery complex aneurysms: anatomic characteristics as predictors of surgical outcome in 300 cases. World Neurosurg.

[9] Brown MA, Parish J, Guandique CF (2017). A long-term study of durability and risk factors for aneurysm recurrence after microsurgical clip ligation. J Neurosurg.

